# Prospective evaluation of PI-RADS v2 and quantitative MRI for clinically significant prostate cancer detection in Indian men – East meets West

**DOI:** 10.1080/2090598X.2022.2072141

**Published:** 2022-05-15

**Authors:** Vijay Kubihal, Vikas Kundra, Vivek Lanka, Sanjay Sharma, Prasenjit Das, Rishi Nayyar, Chandan J Das

**Affiliations:** a Department of Radiodiagnosis and Interventional Radiology, Urology All India Institute of Medical Sciences, New Delhi, India; b Department of diagnostic radiology, MD Anderson Cancer Center, Houston, Texas, USA

**Keywords:** Prostate cancer, Multiparametric MRI, PI-RADS v2, quantitative MRI parameters

## Abstract

**Purpose:**

To validate the detection of clinically significant prostate cancer (Gleason’s score ≥7) by PI-RADS v2 and to assess the ability of quantitative MRI parameters to detect clinically significant prostate cancer (CSPCa) in Indian men.

**Methods:**

Adult men (n = 95) with serum PSA >4 ng/ml were prospectively evaluated with multiparametric MRI (mpMRI) followed by histopathological evaluation using systematic 12-core prostate biopsy in 69 patients and prostatectomy specimens in 26 patients, performed within six weeks of mpMRI. The imaging and the pathology were divided into 12 sectors per prostate. For the validation of PI-RADS v2, a cut-off of PI-RADS v2 score ≥ 3 and PI-RADS v2 score ≥ 4 were compared to histopathology as a reference standard. Further, quantitative parameters, apparent diffusion coefficient (ADC), K^trans^, and K_ep_ were correlated with the Gleason score and evaluated for their ability to distinguish between sectors with CSPCa and sectors without CSPCa.

**Results:**

PI-RADS score ≥ 4 showed higher specificity (89%) than PI-RADS score ≥ 3 (72.2%) at the cost of mild but not significant reduction of sensitivity (sensitivity–87.6% vs 91.9), (n = 1,140 sectors, 95 patients). PI-RADS v2 and quantitative parameters demonstrated the ability to discriminate sectors positive vs negative for CSPCa: AUC (area under the curve) for ADC was 0.928, PI-RADS v2 was 0.903, K^trans^ was 0.897 and K_ep_ was 0.695. Gleason score correlated well with PI-RADS (r = 0.74), ADC (r = −0.73) and K^trans^ (r = 0.69).

**Conclusion:**

PI-RADS v2 is a reliable method for the detection and localization of clinically significant prostate cancer in Indian men, suggesting applicability beyond European or American demographics. Quantitative mpMRI parameters can detect clinically significant prostate cancer with similar test characteristics as PI-RADS v2.

## Introduction

Prostate cancer is the second most common cancer in men [1]. However, in 2021, the estimated number of new cases of prostate cancer in the USA was 248,530, surpassing all other cancers in males, with an estimated death of 34,130, which is next to lung cancers in males [2]. Early diagnosis and timely treatment of clinically significant prostate cancer (CSPCa) can contribute to the improved life expectancy of prostate cancer patients [3]. Serum prostate-specific antigen (PSA) is a commonly used screening parameter for the detection of prostate cancer. However, the lack of specificity associated with serum PSA screening leads to overdiagnosis [4,5] with detection of a higher number of CSPCa, and this often results in overtreatment and accompanying psychological and physiologic consequences. Multiparametric MRI (mpMRI) PI-RADS v2 (2015) was developed to improve the detection, localization, and risk stratification of patients with suspected prostate cancer [6]. A goal of mpMRI PI-RADS score is to aid diagnosis of CSPCa with high sensitivity and negative predictive value [7] and help avoid unnecessary biopsies of the clinically insignificant lesions, and thus guide management. Moreover, mpMRI localizes disease within the prostate, providing targets for biopsy of CSPCa to confirm the diagnosis. Patient demographics can alter the incidence, appearance, and responsiveness of their cancer. Even with standardized acquisition, PI-RADS v2 has shown wide variability among institutions suggesting the potential importance of diversity in PI-RADS score/prostate cancer by ethnicities, such as age at cancer detection, Gleason score, and proportion of patients with metastatic disease [8–10]. Moreover, PI-RADS v2 has been tested primarily in the European and American populations, where incidence is high. Studies in other populations are required to assess the universality of this diagnostic system. That is why we performed this study in Indian men, where the incidence of CSPCa is low, to see differences in presentation, the performance of PI-RADS reads, and biopsy outcomes by patient ethnicity and background.

mpMRI includes T2-weighted imaging, diffusion-weighted imaging (DWI), and dynamic contrast-enhanced (DCE) imaging. PI-RADS v2 uses visual assessment, and thus interpretation can be variable [8]. Both DCE and DWI components can also be evaluated quantitatively. DCE quantitative parameters include volume transfer constant, K^trans^, reflecting efflux of contrast from blood plasma to extravascular, extracellular space, and K_ep_, the transfer rate constant between extravascular, extracellular space, and the blood plasma. Published meta-analysis has shown that visual and quantitative methods of DCE analysis resulted in the same area under the curve (AUC) and DCE-MRI improves AUC for detection of prostate cancer compared with T2-weighted imaging alone [11]. A problem with DCE is that it requires special software for its analysis for quantitative evaluation. DWI can also be quantified using apparent diffusion coefficient (ADC) and meta-analysis has suggested that the ADC measurement appears to improve prostate cancer detection and can be a useful adjunct to conventional anatomic MRI sequences [12]. ADC is preferred to DCE because it is simpler in that it does not require intravenous contrast; however, quantitation of ADC can vary between scanners and institutions. PI-RADS has shown a moderate correlation with Gleason score, a histologic method used to parse low vs high-grade disease [13]. The correlation of quantitative assessments with the Gleason score needs further study in all populations but is particularly lacking in non-European, non-American demographics like the Indian population to understand the universality of the utility of such parameters. Comparison of quantitative parameters to PI-RADS v2 performance is also needed, including in this demographic.

The purpose of this study was to validate the detection of clinically significant prostate cancer in patients with serum PSA > 4.0 ng/ml by PI-RADS v2 and to assess the ability of quantitative MRI parameters to both detect and grade clinically significant prostate cancer in an Indian demographic.

## Materials and methods

This prospective study included consecutive adult men, visiting the department of urology, All India Institute of medical sciences, New Delhi with serum PSA > 4 ng/ml, accrued between December 2016 to November 2018. Serum PSA levels were obtained within a week of MRI. Exclusion criteria included: Non-consenting patients, MRI contraindications such as cardiac pacemakers, contraindications for contrast administration such as the previous history of reaction to Gadolinium, poor general condition/ performance status (e.g.- acute cardiac failure, etc.), history of previous radiation/hormonal/surgical treatment for prostatic diseases.

**Figure 1.**
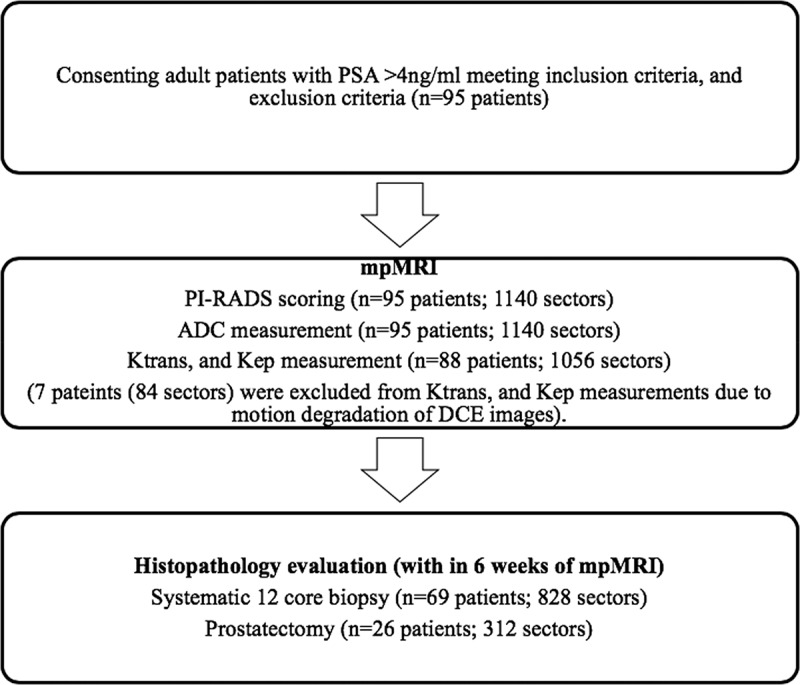
Protocol schema. (PSA – Prostate Specific Antigen; PI-RADS v2: Prostate Imaging Reporting And Data System version 2; ADC: Apparent Diffusion Coefficient; DCE: Dynamic Contrast-Enhanced; mpMRI: multiparametric MRI).

The prospective study (Figure 1) was approved by the institutional ethics committee. Ninety-five consecutive men who fulfilled the inclusion criteria were prospectively evaluated with mpMRI after obtaining written informed consent. Following MRI, 69 men underwent a systematic 12-core prostate biopsy within 6 weeks of mpMRI. In the other 26 patients, prostatectomy was performed within 6 weeks of mpMRI.

### MRI technique

All patients underwent mpMRI on a closed configuration superconducting 3 T MRI system (Ingenia 3 T, Philips, Amsterdam, Netherlands) using an external phased-array body coil. No endorectal coil was used. The MRI protocol (Table 1) consisted of multiplanar conventional MRI sequences including T2 non-fat saturated sagittal, T2 and T1 non-fat saturated axial (thin slice) as well as functional sequences that included DWI, and DCE imaging. DWI was performed with single-shot echo-planar imaging sequences. Four b values were used for DWI (0, 500, 1000, and 1500 s/mm2). For DCE MRI, T1 maps were acquired using flip angles of 5 and 15, followed by a multiphase rapid T1-weighted spoiled gradient-echo sequence. A concentration of 0.1 mmol/kg of MR contrast agent, Gadodiamide was given intravenously into an antecubital vein at the rate of 3 ml/sec using an automatic power injector, followed by a 20 ml saline flush. The temporal resolution of the dynamic scan was 4.7 seconds. The acquisition was repeated 80 times with full coverage of the prostate at each time point. The first 5 acquisitions before the start of intravenous contrast agent injection were used to create a reliable baseline for subsequent analysis.

**Table 1.** Sequence parameters for mpMRI.

MRI Parameters	Sagittal T2 WI	Axial T2 WI	Axial T1 WI	Axial DWI	Axial DCE-MRI
TR (ms)	4750	3715	486	5521	4.0
TE (ms)	100	100	8	75	2.0
Number of slices	36	36	36	27	25
Slice thickness (mm)	3	3	3	4	3.5
Slice gap (mm)	0	0	0	0	0
Field of view (mm)	160 x 160	160 x 160	160 x 160	170 x 178	250 x 250
Matrix	248 x 207	292 x 246	268 x 211	92 x 98	180 x 138
Voxel size (mm)	0.65 x 0.77 x 3	0.55 x 0.65 x 3	0.60 x 0.75 x 3	1.84 x 1.82 x 4	1.39 x 1.81 x 3.5
Phase encoding direction	FH	RL	RL	AP	AP
Number of acquisitions	1	2	2	6	1
Acquisition time (min: seconds)	4: 34	5: 20	4: 55	13:54	6: 14

(mpMRI: multiparametric MRI; TR: repetition time; TE: echo time; DWI: Diffusion-Weighted Imaging; DCE: Dynamic Contrast Enhancement, FH: feet to head; RL: right to left; AP: anteroposterior)

### Image interpretation

**Figure 2.**
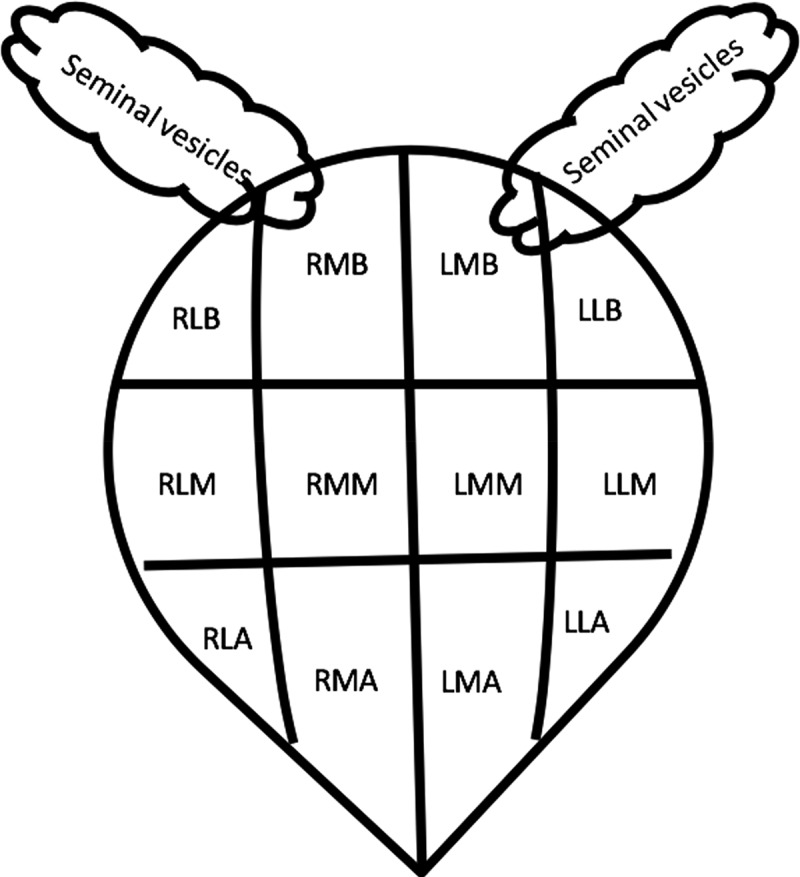
Scheme for the division of prostate into predetermined 12 sectors for the purpose of the study. (right lateral sector at prostate base (RLB), right medial sector at prostate base (RMB), left medial sector at prostate base (LMB), left lateral sector at prostate base (LLB), right lateral sector at mid gland(RLM), right medial sector at mid gland(RMM), left medial sector at mid gland(LMM), left lateral sector at mid gland(LLM), right lateral sector at apex(RLA), right medial sector at apex(RMA), left medial sector at apex(LMA), and left lateral sector at apex(LLA)).

mpMRI images were interpreted by two radiologists (Dr. CJD, 17 years of experience, and Dr. VK, 3 years of experience) in consensus on a PACS workstation (Intellispace Portal 8.0, Philips, Netherlands). Both radiologists were blinded to the final histopathology. For analysis, the prostate was arbitrarily divided into 12 unique sectors as shown in Figure 2. In each of these sectors, PI-RADS v2 score [6] was given by the interpreters for the transitional zone and peripheral zone individually, based on T2 weighted imaging, DWI/ADC, and DCE MRI images, followed by an overall maximum PI-RADS v2 score for the sector. For quantitative analysis of DWI, and DCE MRI, regions of interest (ROI) of areas >10 mm^2^ were drawn in each of the sectors. For measurement of K^trans^, and K_ep_, the ROIs were drawn in each of these sectors, over the region showing maximum abnormality on the respective color map, and the ROI for the arterial input function (AIF) was placed over the iliac vessels. Mean values for K^trans^, K_ep_, and ADC were noted for all the sectors.

### Quantitative parameters

ADC values and quantitative DCE parameters were derived on a PACS workstation. All the b-values were used to derive the ADC values. Quantitative DCE parameters were derived using a standard Tofts tracer kinetics model. Seven patients were excluded from the quantitative DCE imaging analysis due to motion limiting evaluation.

### Histopathology analysis

Histopathology was interpreted by two pathologists in consensus (a senior resident pathologist with 5 years of experience, and a faculty with 35 years of experience). Both pathologists were blinded to the imaging findings. On histopathology, the presence and absence of prostate cancer were noted for each of the sectors, with a corresponding Gleason score for the sector with prostate cancer. Clinically significant prostate cancer (CSPCa) was defined as prostate cancer with Gleason score ≥7(3 + 4).

### Statistical analysis

Statistical analyses were carried out on statistical software (STATA 14.1). Values obtained by the study of each qualitative variable were expressed as frequencies; whereas, continuous variables were expressed as mean ± standard deviation (S.D). For the validation of PI-RADS v2, 2 × 2 contingency tables were drawn separately with PI-RADS ≥ 3 and PI-RADS ≥ 4 as cut-offs, using histopathological findings as the reference standard. Sensitivity, specificity, positive predictive value (PPV), and negative predictive value (NPV) were tabulated. The various quantitative mpMRI parameters (ADC, K^trans^, K_ep_) were evaluated for their ability to distinguish between sectors with CSPCa and sectors without CSPCa using independent samples t-test. Spearman rank correlation was calculated between Gleason score and the above-mentioned quantitative parameters, and PI-RADS v2 score. For all parameters, ROC analysis was carried out to determine their discriminative ability to distinguish biopsy sectors with CSPCa from biopsy sectors with no CSPCa, and a suitable cut-off was also suggested for quantitative parameters based on ROC analysis that had the best sensitivity as well as specificity without significant compromise in either.

## Results

Participant characteristics are shown in Table 2. Of the 1,140 sectors (12 sectors per patient in 95 patients) assessed, 396 sectors (34.7%) were positive for CSPCa.

**Table 2.** Participant characteristics.

Number of patients	95 men
Age: range; mean (SD)	45 to 78 years; 64.9 years (7.2 years)
Serum PSA: range; mean (SD)	5.9 to 161.6 ng/ml; 25.3 ng/ml (23.9 ng/ml)
Prostate volume: range; mean (SD)	13.97 to 175.02 ml; 55.78 ml (33.95 ml)
PSA density: range; mean (SD)	0.10 to 2.62ng/ml^2^; 0.56ng/ml^2^ (0.52ng/ml^2^)
Symptoms	Obstructive LUTS: 90.5% (86/95); Irritative LUTS 1.1% (1/95); Asymptomatic 8.4% (8/95)

(PSA: Prostate Specific Antigen; LUTS: Lower Urinary Tract Symptoms; SD: Standard Deviation; PZ: Peripheral Zone; TZ: Transitional Zone)

### Validation of PI-RADS v2 score

**Figure 3.**
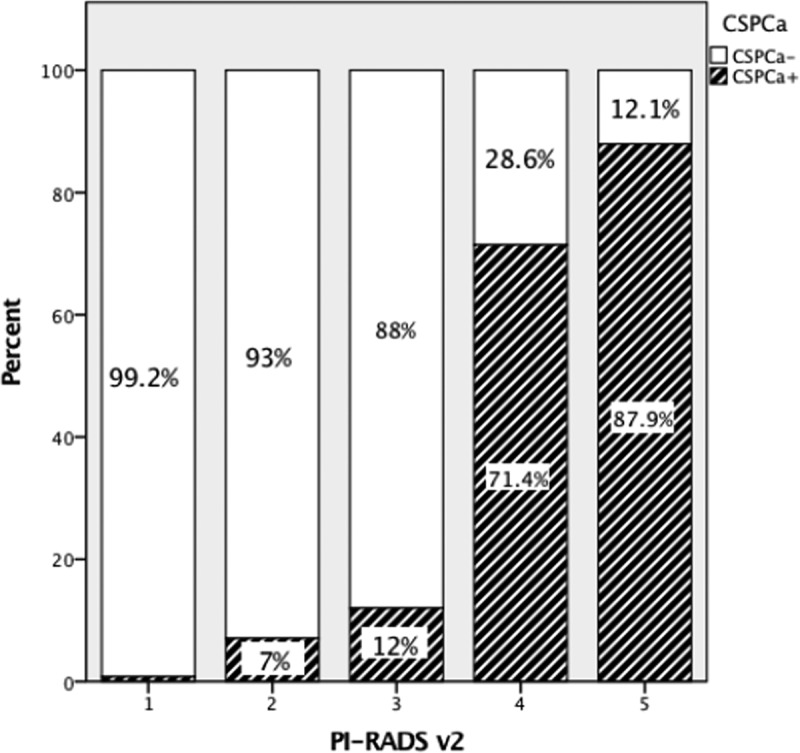
Percentage of sectors with clinically significant prostate cancer identified according to PI-RADS v2 score. (PI-RADS v2: Prostate Imaging Reporting And Data System version 2; CSPCa+: Sectors positive for clinically significant prostate cancer; CSPCa-: sectors negative for clinically significant prostate cancer).

Percentage of sectors showing CSPCa was highest in PI-RADS v2 score 5 (87.9%), with a lower percentage in lower PI-RADS v2 scores (71.4% in PI-RADS v2 score 4; 12% in PI-RADS v2 score 3; 7% in PI-RADS v2 score 2; 0.8% in PI-RADS v2 score 1). Conversely, the percentage of sectors showing no CSPCa was highest in PI-RADS v2 score 1 (99.2%), with a lower percentage in higher PI-RADS v2 scores (Figure 3).

Cut-off of PI-RADS v2 score ≥ 3 resulted in a sensitivity of 91.9% (364/396) (95% CI – 88.8% to 94.4%), specificity of 72.2% (537/744) (95% CI – 68.8% to 75.4%), positive predictive value (PPV) of 63.7% (364/571) (95% CI – 59.7% to 67.7%), and negative predictive value (NPV) of 94.4% (537/569) (95% CI – 92.2% to 96.1%) for detection of CSPCa. Cut-off of PI-RADS v2 score ≥ 4 resulted in a sensitivity of 87.6% (347/396) (95% CI – 84% to 90.7%), specificity of 89% (662/744) (95% CI – 86.5% to 91.1%), PPV of 80.9% (347/429) (95% CI – 76.8% to 84.5%), and NPV 93.1% (662/711) (95% CI – 91% to 94.9%) for detection of CSPCa (n = 1140 sectors, 95patients).

A cut off of PI-RADS score ≥ 4 showed improved overall specificity and PPV than a cut off of PI-RADS score ≥ 3 (p < 0.001 for specificity, 89% vs 72.2%%; and p < 0.001 for PPV, 80.9% vs 63.7%), with mild but not statistically significant reduction in sensitivity (p > 0.05 for sensitivity 87.6% vs 91.9%) and similar NPV for detection of CSPCa (p > 0.05). (n = 1140 sectors, 95patients).

### Quantitative mpMRI parameters

Seven patients were excluded from the analysis of quantitative DCE imaging due to motion limiting evaluation. Hence, only 88 patients (1056 sectors) were available for analysis of quantitative DCE. Quantitative parameter ADC was analyzed in all 95 patients. Table 3 demonstrates the mean value and 95% Confidence Interval (CI) of quantitative MRI parameters, which showed highly significant differences in sectors with and without CSPCa.

**Table 3.** Mean value (95% CI) of quantitative mpMRI parameters (K^trans^, K_ep_, and ADC) in sectors with clinically significant prostate cancer (CSPCa) and sectors without clinically significant prostate cancer (No CSPCa).

Parameters	CSPCa	No CSPCa	P-value
Mean K^trans^ (95% CI) (x 10^−3^ min^−1^)	22.4 (21.84–22.96)	12.6 (12.21–13.17)	<0.001 (n = 1056 sectors)
Mean K_ep_ (95% CI) (x 10^−3^ min^−1^)	409.52 (393.31–425.73)	308.94 (298.16–319.73)	<0.001 (n = 1056 sectors)
Mean ADC (95% CI) (x 10^−3^ mm^2^/sec)	0.67 (0.65–0.68)	1.08 (1.06–1.1)	<0.001 (n = 1140 sectors)

(CSPCa: Clinically Significant Prostate Cancer; ADC: Apparent Diffusion Coefficient; CI: Confidence Interval)

**Figure 4.**
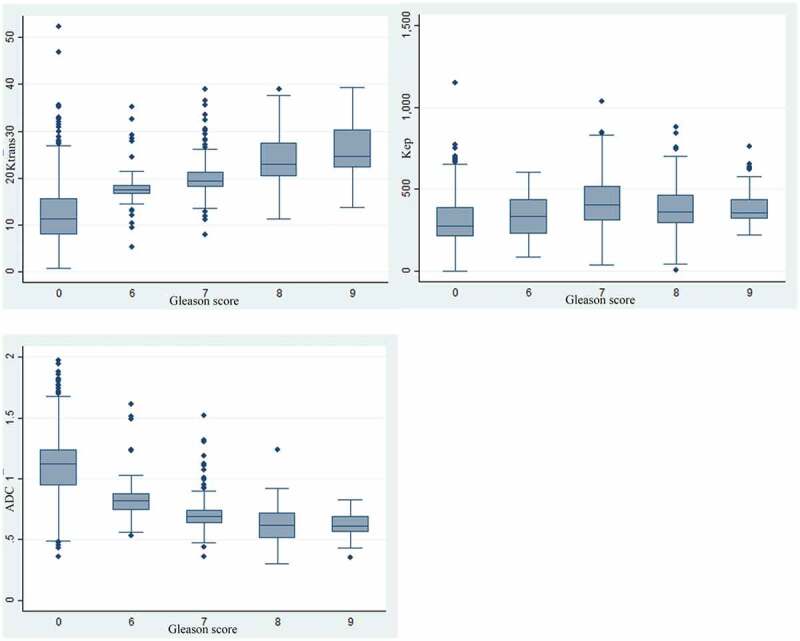
Box plots showing the distribution of K^trans^(4a), K_ep_(4b), and ADC(4c) against the Gleason score. (ADC: Apparent Diffusion Coefficient).

Spearman rank correlation co-efficient (r) between Gleason score and PI-RADS v2 as well as quantitative mpMRI parameters K^trans^, K_ep_, and ADC showed strong correlation with PI-RADS v2 (positive correlation, r = 0.74, p-value <0.001, n = 1140 sectors (95 patients)), and ADC (strong negative correlation, r = −0.732, p-value <0.001, n = 1140 sectors (95 patients)), whereas K^trans^ correlation was moderate (positive correlation, r = 0.69, p-value <0.001, n = 1056 sectors (88 patients)). K_ep_ showed weak correlation with Gleason score (r = 0.307, p-value <0.001, n = 1056 sectors (88 patients)). Figure 4 demonstrates box plot showing distribution of K^trans^, K_ep_, and ADC against Gleason score. Note that these correspond with Spearman rank analysis. Note also that the error bars overlap among the different Gleason scores.

**Figure 5.**
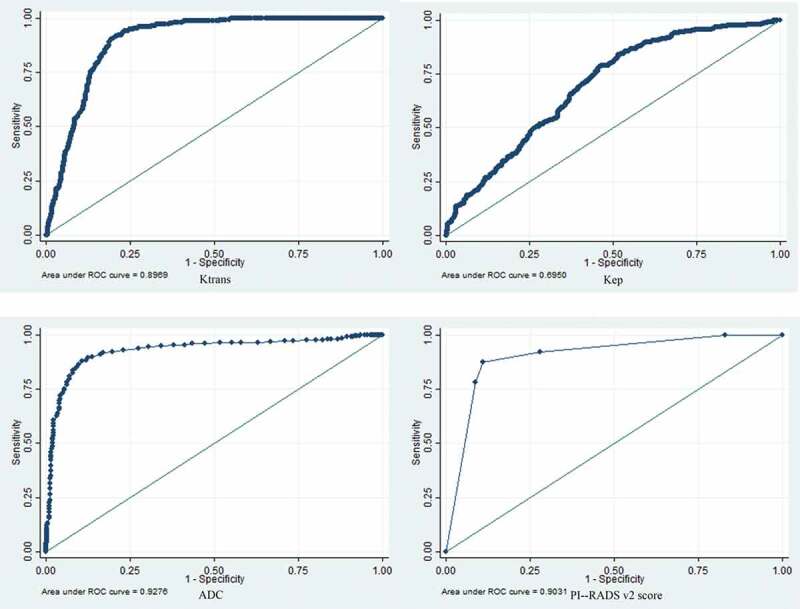
ROC curves of K^trans^(5a), K_ep_(5b), ADC(5c), and PI-RADS v2 score(5d). (ADC: Apparent Diffusion Coefficient; PI-RADS v2: Prostate Imaging Reporting And Data System version 2).

**Figure 6.**
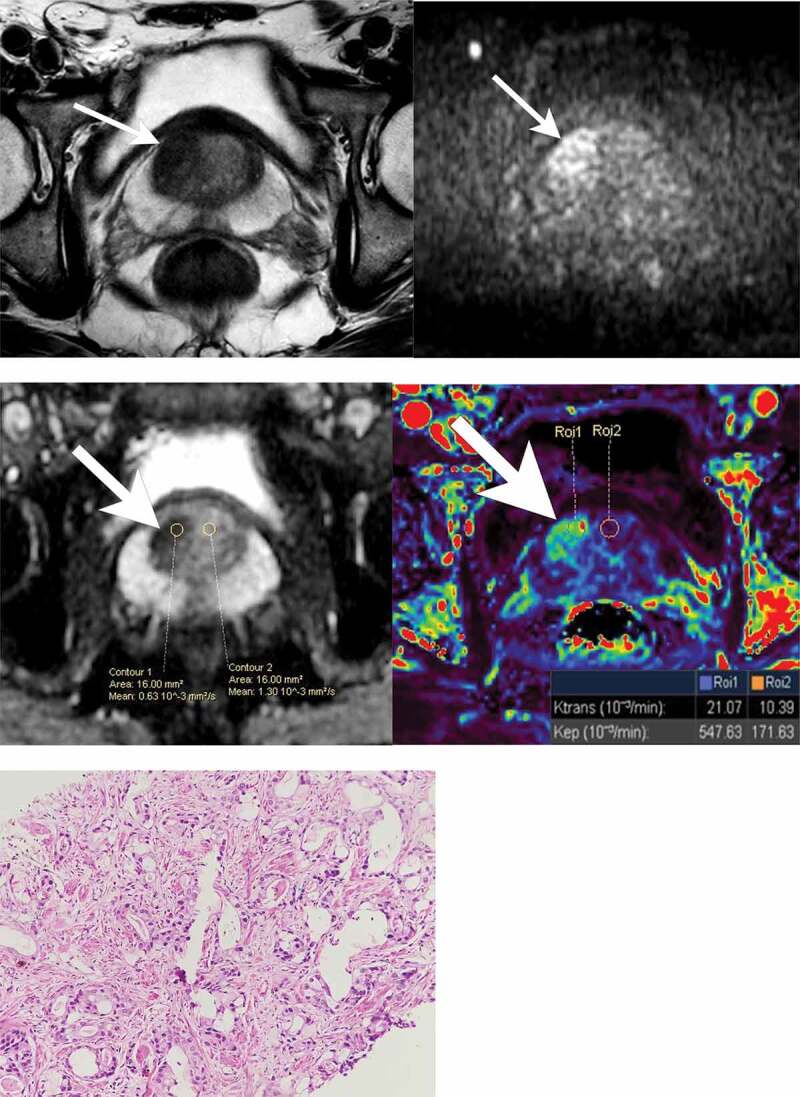
PI-RADS v2 score 4 lesion, and corresponding quantitative MRI parameters. 63 year old man who presented with obstructive lower urinary tract symptoms and elevated PSA (17.7 ng/ml). MRI showed PI-RADS 4 lesion in the right medial sector of the prostate base. Non-fat suppressed axial T2 weighted MR image (6a) shows a lenticular shaped hypointense lesion in the right medial sector of the prostate base (in the anterior transitional zone), with a maximum diameter of less than 1.5 cm, and diffusion restriction on High b-value (1500 sec/mm^2^) diffusion-weighted trace axial image (6b) and corresponding ADC map (6c). DCE K^trans^ color map (6d) shows a significant abnormality in the lesion. ROIs drawn over the lesion (histologically proven clinically significant prostate cancer) in the right medial sector, shows ADC (6c), K^trans^, and K_ep_ (6d) of 0.63 × 10^−3^ mm^2^/sec, 21.07 x 10^−3^/min and 547.63 x 10^−3^/min, respectively. ROIs drawn in the left medial sector with normal MRI (and no prostate cancer on histopathology) shows ADC (6c), K^trans^, and K_ep_ (6d) of 1.30 × 10^−3^ mm^2^/sec, 10.39 x 10^−3^/min and 171.63 x 10^−3^/min respectively. Note that clinically significant prostate cancer in the right medial sector shows lower ADC value, and higher K^trans^ and K_ep_ values, compared to the sector without clinically significant prostate cancer. 6e) Corresponding histopathology from the right medial sector showed clinically significant prostate cancer with a Gleason score of 7 (4 + 3).

Table 4 summarizes the ROC curve analysis, with AUCs, optimal cut-offs, sensitivity, and specificity of K^trans^, K_ep_, and ADC, for detection of CSPCa. ADC showed the highest discriminative ability (AUC – 0.928) with suggested optimal cut-off of 0.8 mm^2^/sec, at which sensitivity and specificity were 88.1% (95% CI – 84.5% to 91.1%) and 88.3% (95% CI – 85.8% to 90.5%) respectively. Figure 5 shows ROC curves of quantitative parameters. Figure 6 is an illustrative example of PI-RADS v2 score 4 lesion, and corresponding quantitative MRI parameters (ADC, K^trans^, and K_ep_).

**Table 4.** ROC curve analysis to determine the discriminative abilities of K^trans^, K_ep_, and ADC for detection of clinically significant prostate cancer.

Parameters	ADC	K^trans^	K_ep_	PI-RADS v2
AUC (95% CI)	0.928 (0.911–0.944)	0.897 (0.878–0.916)	0.695 (0.663–0.727)	0.903 (0.885–0.921)
Optimal cut-off	0.80 x 10^−3^ mm^2^/sec	17.9x 10^−3^ min^−1^	334 x 10^−3^ min^−1^	PI-RADS v2 score 4
Sensitivity (95% CI)	88.1% (84.5% – 91.1%)	83.2% (78.9% – 86.9%)	63.9% (58.7% – 68.9%)	87.6% (84% – 90.7%)
Specificity (95% CI)	88.3% (85.8% – 90.5%)	83.5% (80.6% – 86.2%)	63.1% (59.3% – 66.7%)	89% (86.5% – 91.1%)

(ROC: Receiver Operating Characteristic; ADC: Apparent Diffusion Coefficient; PI-RADS v2: Prostate Imaging Reporting And Data System version 2; AUC: Area Under Curve; CI: Confidence Interval)

## Discussion

mpMRI PI-RADS v2 detected and localized CSPCa in this non-European, non-American patient demographic of Indian men; this supports the universality of PI-RADS v2 in diverse patient populations. We noted that the percentage of sectors with CSPCa was greater with a higher PI-RADS v2 score using a sector-based approach. This is supported by similar findings by Kasivisvanathan et al. [14] using a more general patient-based approach. A cut-off of PI-RADS v2 score ≥ 4 showed significantly higher specificity and importantly PPV for detecting CSPCa than a cut-off of PI-RADS score ≥ 3 supporting the numerical system for expressing confidence in detecting prostate cancer, with a mild insignificant reduction in sensitivity. PI-RADS and quantitative mpMRI parameters, ADC, and K^trans^ correlated with Gleason score and thus there is an association with tumor aggressiveness in addition to detecting CSPCa; however, there was overlap between the quantitative parameters and Gleason score and we cannot say if these parameters predict aggressiveness for a particular individual. We found that ROC analysis of quantitative MRI parameters for detection of CSPCa showed high discriminative ability for quantitative ADC (AUC – 0.928) and K^trans^ (AUC–0.897) similar to PI-RADS v2 (AUC- 0.903) and given the variability in PI-RADS interpretation among readers and institutions [8], we suggest that quantitative parameters have potential to add value by decreasing such variability.

Our PI-RADS v2 results (sensitivity and specificity of 92% and 72% for PI-RADS ≥3 and 88% and 89% for PI-RADS ≥4 respectively) compare favorably to prior studies using radical prostatectomy/systematic biopsies as reference standards; of note, prior studies have reported wide variation in PI-RADS v2 scoring for detection of prostate cancer with sensitivity ranging from 44% to 93% and specificity ranging from 38% to 94% [15–24]. Variability has also been reported for strengths of correlation and discriminative potentials (AUCs of ROCs) for each of the quantitative MRI parameters, ADC, K^trans^, and K_ep_) [25–36]. These studies showed a correlation coefficient between Gleason score and quantitative MRI parameters ranging between 0.38 and 0.62 for K^trans^; 0.30 and 0.77 for ADC; and 0.13 and 0.43 for K_ep_. AUC for ROC among these studies for quantitative MRI parameters for detecting CSPCa ranged from 0.59 to 0.77 for K^trans^; 0.61 to 0.76 for k_ep_; and 0.69 to 0.81 for ADC. The observed heterogeneity may be explained by the varied study designs and definitions of prostate cancer (i.e. include or exclude Gleason 6), acquisition protocols, different cut-offs, biopsy inaccuracies, user experience with the PI-RADS v2 system, differences in pharmacokinetic models used for generating K^trans^ and K_ep_ maps, and differences in the b-values and model fitting used to construct ADC maps. Thus, standardization is needed. Even with standardized acquisition, PI-RADS v2 has shown wide variability among institutions suggesting the potential importance of diversity in PI-RADS/prostate cancer by ethnicity [8,9]. The clinical features from different racial groups differed significantly at the time of newly diagnosed PCa with a report suggesting PCa in Hispanic men is generally more aggressive at diagnosis [9]. Associations of race/ethnicity and PI-RADS score with risk of PCa or CSPCa have been reported by Hines et al. as they found nonHispanic Black (NHB) men have higher odds for overall PCa and CSPCa compared to nonHispanic White (NHW) men [10]. These features probably explain the differences in presentation, PI-RADS reads, biopsy outcomes by patient ethnicity, and background in the Indian population with a low incidence of prostate cancer. In our study, the diagnostic accuracy of PI-RADS v2 scoring, and correlation of quantitative parameters with Gleason score was comparable to or better than that of the above-mentioned studies. We also found that K^trans^ and ADC had better discriminative ability to detect clinically significant prostate cancer than K_ep_. Findings suggest that quantitative parameters like K^trans^ and ADC have a potential role in detecting CSPCa and are associated with Gleason score thus potentially with tumor aggressiveness. Therefore, they may aid in risk stratification, which needs to be formally tested. Standardization of technique, software, and interpretation methods should increase homogeneity in interpretation.

We found higher PPV (64%) than the PPV interquartile ranges reported by Westphalen et al. for multiple institutions (27–48%) for PI-RADS ≥3 and higher PPV (81%) than interquartile range (34–65%) for PI-RADS ≥4 [8], which may be contributed to by not only technique but also patient demographics. They also noted PPV confidence interval ranges of 27% – 43% for PI-RADS ≥3 and 40% – 58% for PI-RADS ≥4 [8]. Jordan et al. reported an area under the ROC curve of 0.69 (95% CI 0.63–0.76) for PI-RADS v2 in the non-academic setting suggesting it is a strong predictor of CSPCa [37]. Thus, there remains wide variability among centers in PI-RADS test performance even after acquisition and interpretation using PI-RADS recommendations. We found that ADC and K^trans^ AUC was similar to that of PI-RADS v2 and we suggest that standardized quantitative parameters may be used to incorporate or replace some PI-RADS criteria to increase test reproducibility; this would need to be formally tested. The use of standardized or quantitative methods to localize prostate cancer in the prostate may help in avoiding overdiagnosis and management of CSPCa and at the same time, it may help in the accurate selection of targets for guiding biopsies and focal ablation therapies.

Limitations of our study include that 1) the majority of our patients had prostate biopsy as the histopathological reference standard. Prostate biopsy is subjected to the risk of sampling error, making it a less robust reference standard than serially sectioned radical prostatectomy specimens. 2) For those patients assessed with prostatectomy specimens as the histological reference standard, we cut each of these axial sections into smaller sectors as described in the methodology. However, due to the lack of whole-mount histopathological sections, a small possibility of a mismatch between MRI sectors and histopathological sectors remains. 3) In this study, we did a sector-based analysis. The transitional zone and peripheral zone in the respective sector were evaluated as one, and not separately. 4) PI-RADS v2 was used, instead of the more recent PI-RADS v2.1; however, changes in interpretation by PI-RADS v2.1 are mainly in lower PI-RADS scores (score 1 and score 2) of the transitional zone, which are mostly not cancer. 5) Use of DWI with multiple b values and a high in-plane resolution as per strict adherence to PI-RADS v2 guidelines has increased the sequence time to over 13 min. This may cause motion artifacts; which were seen in seven of our patients which limited quantitative evaluation. 6) We did not evaluate quantitative analyses over multiple scanner types; reproducibility across scanners and between centers still needs to be addressed in the field.

## Conclusion

mpMRI PI-RADS v2 detected and localized clinically significant prostate cancer in this non-European, non-American patient demographic of Indian men, supporting the universality of PI-RADS v2 in diverse patient populations. Quantitative mpMRI parameters ADC and K^trans^ are associated with Gleason score and can detect CSPCa with similar high AUC test characteristics as PI-RADS v2; this leads to a hypothesis to be tested that standardized quantitative mpMRI measures could be potentially used to improve interobserver variability in PI-RADS interpretation.

## Abbreviations

PI-RADS v2: Prostate Imaging Reporting And Data System version 2; CSPCa: Clinically Significant Prostate Cancer; mpMRI: multiparametric Magnetic Resonance Imaging; PSA: Prostate Specific Antigen; ROI: Region of Interest; ADC: Apparent Diffusion Coefficient; DWI: Diffusion-Weighted Imaging; DCE: Dynamic Contrast-Enhanced; ROC: Receiver Operator Curve; AUC: Area Under Curve

## Author contibutions

- Conceptualization: Vijay Kubihal, Vivek lanka, Chandan J Das, Sanjay Sharma, Prasenjit Das, Rishi Nayyar
- Data curation: Vijay Kubihal, Vivek lanka
- Formal analysis: Vijay Kubihal
- Funding acquisition: N/A
- Investigation: Vijay Kubihal, Chandan J Das
- Methodology: Vijay Kubihal,, Vivek lanka, Chandan J Das
- Project administration: Vijay Kubihal,, Vivek lanka, Chandan J Das
- Resources: Vijay Kubihal, Chandan J Das, Sanjay Sharma, Prasenjit Das, Rishi Nayyar
- Software: Vijay Kubihal
- Supervision: Vijay Kubihal, Chandan J Das, Vikas kundra
- Validation: Vijay Kubihal, Chandan J Das
- Visualization: Vijay Kubihal, Chandan J Das, Vikas kundra
- Writing-original draft: Vijay Kubihal, Chandan J Das, Vikas kundra
- Writing-review & editing: Vijay Kubihal, Chandan J Das, Vikas kundra, Sanjay Sharma, Prasenjit Das, Rishi Nayyar
